# High-Density Dermal Matrix for Soft Tissue Augmentation Using a Matrix Tissue Graft Technique—A Comprehensive Multicenter Analysis of 20 Implants: A 1-Year Follow-Up Retrospective Study

**DOI:** 10.3390/jcm13102954

**Published:** 2024-05-17

**Authors:** Alessandro Minniti, Marino Caroprese, Morris Zarantonello, Daniele De Santis, Gialfonso Caliendo, Federico Gelpi

**Affiliations:** 1 Independent Researcher, 07100 Sassari, Italy; alessandrogavinominniti@gmail.com; 2 Department of Clinical and Experimental Medicine, University of Foggia, 71122 Foggia, Italy; marinocaroprese@virgilio.it; 3 Head and Neck Department, Surgery, Dentistry, Pediatrics and Gynecology, University of Verona, 37129 Verona, Italy; morris.zarantonello@hotmail.it (M.Z.); daniele.desantis@univr.it (D.D.S.); 4 Department of Diagnostics & Public Health, Specialization School in Health Statistics and Biometry, University of Verona, 37129 Verona, Italy; gialfonso@hotmail.it

**Keywords:** soft tissue augmentation, biomechanics, high-consistency dermal matrix, scaffold-like structure, periodontal surgery, full-thickness flap, minimal invasiveness, intraoral scan, digital flow

## Abstract

**Background**: In this multicenter case series analysis, the authors present successful instances of 20 single-screw-retained and implant-supported prosthetic rehabilitation samples. **Methods**: A high-density heterologous dermal matrix (Derma^®^ Osteobiol by Tecnoss, Torino, Italy) was employed with a specific technique named the matrix tissue graft (MTG) in all these cases characterized by an inadequate initial supra-crestal tissue height (thin if 1 mm or medium if 2 mm) to enhance the peri-implant soft tissues both vertically and horizontally. **Results**: The implants were deemed successful in all cases, yielding a success proportion of 100% (one-sided 97.5% confidence interval = 83.2–100%). The buccal and lingual gains were, respectively, 2.2 ± 0.38 mm (range 1.7–3.22 mm) and 0.83 ± 0.33 mm (range 0.1–1.5 mm). These measurements were calculated as the maximum distance between two superimposed .stl file models (derived from two different IOS devices) scanned before implant placement and 1 year after dermal matrix healing. **Conclusions**: An outstanding vertical and horizontal gain was obtained using this heterologous derma matrix placed above the bone crest and surrounding the dental implants.

## 1. Introduction

**Figure 1:**
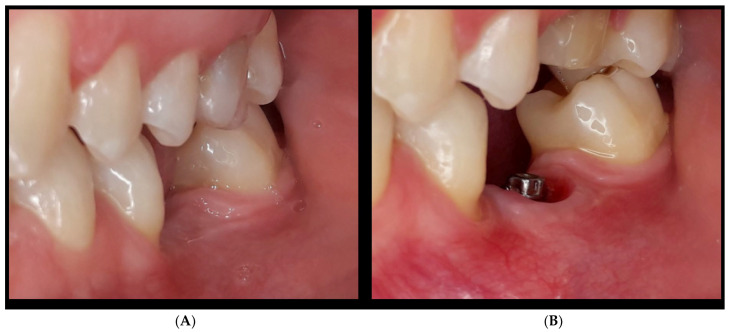
(**A**) intraoral aspect of the vertical and horizontal deficiency of soft tissue in the edentulous ridge; (**B**) the same view after 1 year of soft tissue healing: the new soft tissue profile is appreciable due to a dermal matrix application.

At present, the surge in interest in aesthetic dental procedures among patients has heightened the focus on periodontics and implant prosthetics. This emphasis extends to managing gum recession in natural teeth and restoring insufficient supra-crestal tissue heights around dental implants. Periodontal surgery emerges as a pivotal solution for rectifying the defects and deficiencies in periodontal tissues, with the integration of innovative approaches such as the connective tissue graft technique and the use of a high-consistency dermal matrix with a 2 mm thickness (Derma^®^ Osteobiol by Tecnoss, Torino, Italy) providing advanced solutions for soft tissue management in periodontal care [1,2] (Figure 1).

In a recent article, the authors introduced an inventive approach to enhance the horizontal and vertical volume of crestal and supra-crestal soft tissues. This technique employs a specific high-consistency dermal matrix, setting it apart from other autologous tissue methods. Notable for its reduced invasiveness, it distinguishes itself from both autologous and techniques involving heterologous or synthetic tissue for two primary reasons: the matrix is strategically positioned beneath a full-thickness muco-periosteal flap, in direct contact with the bony crest, and it eschews fixation aids, being enveloped within the muco-periosteal complex [3].

Advancements in tissue augmentation and expansion techniques and the integrating of biomaterials have marked the substantial progress in periodontal surgery. These innovations have ushered in a profound transformation to the landscape of soft tissue management [3,4,5,6].

Integral to cosmetic and reconstructive procedures, soft tissue administration is pivotal in restoring and enhancing tissue volume, contour, and function. However, conventional methods utilizing synthetic or autologous grafts may be associated with complications, limited graft availability, or donor site morbidity [7]. Steering away from conventional approaches, which always represent a valid alternative for tissue enhancements around teeth and implants [8], the high-consistency dermal matrix has emerged as a leading contender, showcasing promising possibilities for tissue augmentation and distraction within periodontal surgery [1,2,8,9].

The high-consistency dermal matrix has many advantageous attributes for soft tissue grafting. Its scaffold-type architecture provides a 3D multi-dimensional structure that encourages cellular ingrowth and tissue regeneration, facilitating its smooth integration with surrounding tissues and yielding natural and enduring outcomes. Despite the growing interest in this cutting-edge biomaterial, comprehensive multicenter clinical studies evaluating its effectiveness, safety, and long-term outcomes in tissue augmentation and distraction for soft tissue implantations within periodontal therapies remain scarce [1,2,7,8,9].

The main goal of this study was to evaluate, over a 1-year follow-up, the efficacy and stability of an innovative technique utilizing a dermal matrix, attaining the most favorable results in soft tissue augmentation for implant applications characterized by a low-invasive surgical approach.

Additionally, the paper highlights potential areas for subsequent investigations, offering valuable insights for clinicians and researchers keen on employing a high-density dermal matrix for surgical enhancements in periodontal and implant cases.

## 2. Materials and Methods

All team members designed and conceived this retrospective multicenter study with an enrolled sample of 20 bone-level implants placed in 18 partially edentulous patients.

This study was performed in two different clinical offices:

(1) Dr. Minniti Alessandro, Sassari, Italy;
(2) Dr. Gelpi Federico Albaro di Ronco All’Adige, Verona, Italy.

Only three types of the bone level implants were employed, to not introduce further variables:

(1) B1 by IDI evolution Concorezzo MB—Italy;
(2) Dental Tech Active by Dental Tech S.r.l. Misinto MB—Italy;
(3) Cono-in by 3P Smart devices Scarlenghe TO—Italy.

This study was conducted according to the principles of the Helsinki Declaration of 1975, revised in 2000 for biomedical research involving human subjects. Since the authors analyzed preexisting and non-identifiable data from the patients, who were all informed about the nature of the data’s treatment and provided written consent before participation, the proposed retrospective study did not require approval by the review board.

All the 18 patients enrolled in this retrospective study, in terms of their medical history, did not show contraindication to implant surgery: only 1 was a mild smoker (less than 5 cigarettes a day).

The gingival biotype of each patient was diagnostically determined after measuring the distance between the bone level of the edentulous site in the 3D CBCT dicom file and the free gingival margin at the corresponding site, derived from the Stl file. This was possible only through a superimposition of the 3D CBCT dicom files (derived from Cs 8100 Carestream^®^ 3D CBCT and a Planmeca Proface^®^ 3D CBCT devices, Helsinki, Finland) and the intraoral scansions derived from IOSs (Cs 3700 flow Carestream^®^ Rochester, NY, USA and Trios 5 3 Shape^®^ Copenhagen, Denmark).

The radiation dose for each patient was as low as 600 mSy, with FOV 5 × 5 cm^2^.

**Figure 2:**
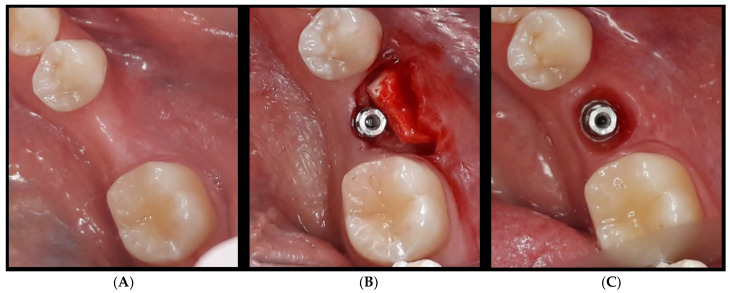
(**A**) occlusal aspect of the edentulous ridge before dermal matrix application: the concave line defined by the soft tissue is appreciable; (**B**) occlusal aspect of the raising flap (with preservation of the papilla) necessary to place the dermal matrix; (**C**) the final buccal gain and new gingival profile after crown unscrewing.

After this initial diagnostic classification, a dermal matrix application would be indicated only in the case of a thin biotype (we mean a supra-crestal height of 1 mm) or medium biotype (we mean a supra-crestal height of 2 mm) (Figure 2).

The residual bone volumes were sufficient and suitable for standard implant insertion without further procedures. Hence, the patient was offered a double treatment involving the insertion of an endo-osseous implant and the simultaneous thickening of their crestal and supra-crestal soft tissues, which were insufficient for a suitable prosthetic rehabilitation from a functional and aesthetic point of view. Once the informed consent was obtained and signed by the patient for the aforementioned proposed treatment, they were provided with hygiene and dietary instructions to follow before, during, and after the implant–prosthetic treatments.

Moreover, the main periodontal indices were marked after the professional oral hygiene session. Once an absence of clinical signs attributable to periodontal disease was detected, the patient was prescribed antibiotic therapy using amoxicillin + clavulanic acid tablets of 1 g, to be taken every 12 h (2 in a day) starting from the day before the operation and to be taken for a further 5 days at the same frequency. Therapy with chlorhexidine 0.12% mouthwash was also administered once a day, to be performed 1 day before the operation and followed for a further 2 days.

The surgery involved the administration of loco-regional anesthesia with articaine 4% infiltration (adrenaline 1:100,000). The full-thickness muco-periosteal surgical flap was prepared using a 15c blade. The main incision extended from the center of the ridge, dividing the keratinized mucosa present into two equal portions, lingual and vestibular. The flap was then perfected through full-thickness medial and distal relief incisions from the primary ones, with a divergent trend and without affecting the papillae of the adjacent elements, reaching the muco-gingival line. Before the bone drilling, a measurement of the supra-crestal height was taken with a periodontal probe (Michigan Periodontal Probe) to confirm the classification of each patient. All patients displayed a supra-crestal tissue probing of 1 or 2 mm, and these biometric parameters served as an indication for the MTG technique.

An osteotomy preparation with progressive drills was performed, and the implant was inserted with an implant micro-motor recording a peak torque of 55 N/cm^2^. A trans-mucosal healing screw with a diameter of 3.8 mm was then positioned on the implant. The vestibular detachment of the full-thickness flap between the two relief incisions was stretched gently with anatomical forceps to check there was sufficient space for the matrix and make sure it was suitable in terms of length and width for its consequent covering and stability.

The thick dermal matrix MTG was separately stored with the greatest possible sterility, adequately shaped, and then positioned without any fixation aids.

The flap was then sutured using a 4/0 PTFE thread, a 16 mm taper cut needle, and simple detached stitches without placing other protective structures, such as membranes. No immediate prosthetic loading was performed.

**Figure 3:**
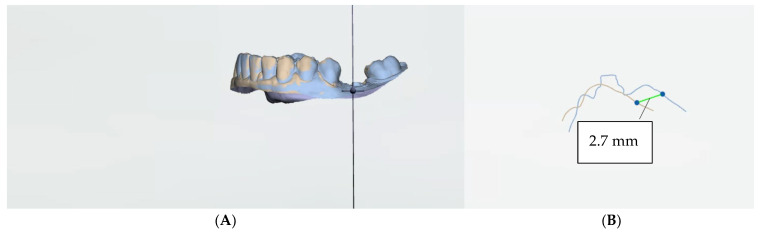
(**A**) The superimposition of T0 and T1 STL files to calculate the buccal gain using dedicated software (Patient Monitoring by 3Shape). (**B**) The distance between the two different-colored lines represents the effective buccal gain due to the application of the dermal matrix.

**Figure 4:**
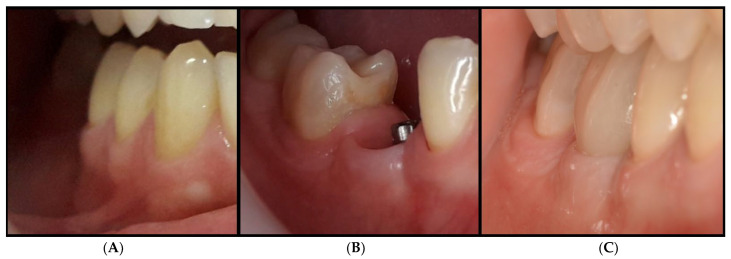
(**A**) The initial buccal aspect of the case: the concave line of the soft tissue accurately indicates the MTG technique; (**B**) the new soft tissue profile after dermal matrix application and soft tissue healing; (**C**) the aesthetic result and functional integration of the dermal matrix with the final restoration and prosthesis delivery.

While waiting for healing, the suture was removed on the 10th day following the operation. Once bone integration had taken place, the first clinical/optical control scan of the soft tissues was performed. After a complete maturation of the tissues, in our opinion, detected at 3 months, a temporary prosthesis for the implant was added, using a PMMA temporary abutment for the single-screw-retained prosthesis. After 30 days, an optical impression was taken to obtain another control measurement and create the definitive prosthetic product, which was positioned 15 days later. The almost complete maturation of the tissues, with stabilization and tissue increase (both horizontal and vertical), was evident at the 6-month and 1-year follow-ups, with a definitive crown. A further clinical control scan is expected in 2 years. The scans obtained were overlapped and compared using image superimposition software (tool ‘Patient Monitoring’ by 3Shape, version 24.1, Copenhagen, Denmark) (Figure 3 and Figure 4).

## 3. Results

### 3.1. Patients’ Characteristics

The case series comprised 18 patients: 5 men and 13 women. The mean age (±SD) was 50.9 ± 8.8 years (33.3–64.5 years). Only 1 patient out of the 18 was a current smoker, and all the patients revealed no contraindications for implant surgeries.

### 3.2. Surgical Technique

Regarding implant type, IDI evolution was used in two thirds of cases (n = 13), while Dental Tech AT and the 3P Smart device were used in a minority of patients (n = 4 and n = 3, respectively).

The mean implant diameter (±SD) was 3.88 ± 0.48 mm (range 3.2–5.5 mm), and the mean length was 10.3 ± 1.6 mm (range 8.14 mm). Regarding the depth of the implant within the tissue, the implant reached a bone level in all cases.

### 3.3. Outcomes

**Figure 5:**
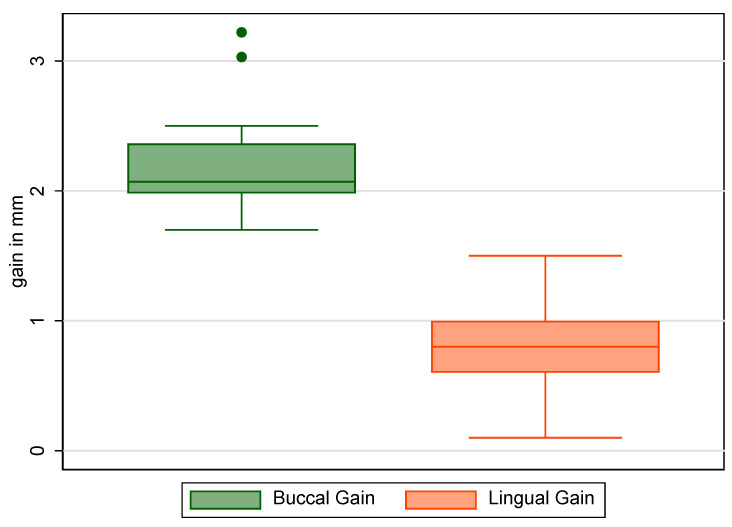
Distribution of buccal and lingual gains, synthesized through box-and-whisker plots. The buccal and lingual gains were, respectively, 2.2 ± 0.38 mm (range 1.7–3.22 mm) and 0.83 ± 0.33 mm (range 0.1–1.5 mm).

Implants were deemed successful in all cases, yielding a success proportion of 100% (one-sided 97.5% confidence interval = 83.2–100%). The buccal and lingual gains were, respectively, 2.2 ± 0.38 mm (range 1.7–3.22 mm) and 0.83 ± 0.33 mm (range 0.1–1.5 mm) (Figure 5).

There is greater gain on the buccal aspect due to the application of the dermal matrix on the buccal side; however, the reduced gain on the lingual aspect depends on the portion of the matrix positioned at the interproximal level.

## 4. Discussion

MTG has proven to be an effective procedure to enhance the horizontal and vertical volumetric growth of soft tissues at the crestal and supra-crestal levels by using a specific dermal matrix with elevated viscosity [3]. It deviates from the conventional methods of tissue thickening using autologous connective tissue due to its reduced invasiveness (eliminating the need for any tissue samples). Furthermore, it distinguishes itself from approaches involving heterologous or synthetic tissue for two primary reasons in particular: the matrix is placed beneath a periosteal mucosal flap of full thickness, making direct contact with the bone crest on one side and with the periosteum of the flap on the other. Secondly, the matrix is not anchored with any fixation aid but is instead carefully positioned following a thorough preparation of the flap [10,11,12].

Thus, the morbidity associated with multiple complex operations is significantly diminished, eliminating the need for sampling while avoiding additional challenges related to positioning and stabilizing the membrane.

The proposed technique allows for vertical volume increases that are superior to traditional CTG, where autologous connective tissue should be thickened before U-shaped suturing.

The clinical gain we obtained with this dermal matrix is not quantitatively matched by other autologous sampling techniques reported in the literature [13,14].

The MTG matrix is distinguished by its elevated consistency, enabling steadfast volumetric support during the initial surgical phase of soft tissue healing induced by the trauma of implant insertion. The current emphasis in implant–prosthetic regeneration for medium- and long-term maintenance and success revolves around the augmentation of peri-implant soft tissues, considering site-specific biotype variation. Typically, this augmentation involves connective or epithelial-connective grafts, which remain the gold standard but come with certain drawbacks, such as the need for a donor site, increased invasiveness, multiple operations, and heightened surgical skills due to added procedural complexities. The high-thickness dermal matrix is applied promptly after implant placement, adhering to prosthetic and biologically guided principles. It is positioned precisely 4 mm more apically than the free margin of the flap, aiming to prevent the documented para-physiological peri-implant resorption seen in cases where the soft tissue thickness measures 2 mm or less [15,16,17].

The significant distinction between a connective tissue graft and a dermal matrix graft lies in the fact that the former, when executed correctly, rapidly co-participates in supra-crestal vascularization. In contrast, the dermal matrix only becomes involved in vascularization after its total integration, which occurs approximately 4–5 months post-placement. As a result, we cannot directly counteract inherent peri-implant bone resorption. Instead, proactive management involves moving the premeditated apical graft positioning by 4 mm, as described earlier.

When the thickness surpasses 3 mm, the implant can be placed in a juxta-CRESTAL position. A distinctive feature is that the MTG is situated beneath a full-thickness flap. In cases where the inter-implant distance exceeds 10 mm, a trapezoidal flap can be employed while preserving the papillae. In cases where the distance is less than 10 mm, it is advisable to incorporate the papillae of adjacent entities within the flap itself, trying to preserve the lingual–palatal aspect of these papillae in every case. The relief incisions of the flap should extend in the coronal–apical direction up to the muco-gingival line. Meanwhile, the crest incision is designed to preserve the keratinized gingiva by dividing it into two portions: the first on the lingual–palatal side and the second on the vestibular side. The matrix should be appropriately sized to be easily accommodated within this flap. Simultaneously, it should be effectively stabilized by the flap without the need for any additional means of fixation. The flap and simple detached stitches contribute to stabilizing the matrix. In specific situations where the muco-gingival line is positioned more apically and the drains are in proximity, simple detached stitches may also be employed on the mesial and distal drains.

The matrix can be situated directly above the implant itself in an occlusal overturned position, which particularly applicable in cases involving a submerged fixture over a healing screw. The MTG can be customized to meet trans-mucosal requirements by forming two portions that encircle the healing screw, one on the mesial side and one on the distal side.

The sutures are typically removed after 14 days. It is crucial to inform the patient about proper postoperative behavior, particularly regarding chewing food, with a preference for reduced consistency or semi-liquid options. Complete epithelialization occurs within 28–35 days, even in areas that may have been left exposed. This represents a significant advantage compared to traditional matrices or membranes, where such occurrences often lead to the complete failure of the entire operation. Furthermore, after 4 months, integration of the matrix is achieved. During this healing period, temporary prosthesis phases can be initiated, starting as early as the second month. The codification of this specific technique was facilitated through STL superimpositions. Indeed, our demonstration involved superimposing pre- and post-treatment intra-oral scans (CS 3700 Flow Carestream—Trios 5 3 Shape) using dedicated software (Patient Monitoring 3 Shape) at T0 before implant placement and T1, 1 year after implant placement (intraoral scanning with prosthesis unscrewed). Each patient’s starting height of their crestal soft tissues was measured at the incision point using a Michigan probe perpendicular to the crest (1 and 2 mm). Precise matching is crucial to prevent data distortions; all the selected cases were marked by single edentulism and stable conditions in the adjacent dental elements. The results revealed the maintenance of tissue volume with a favorable change in thickening and site-specific biotype attributable to the application of the matrix itself. As described earlier and demonstrated in this case series, the MTG technique is aesthetically significant as it restores soft tissue thickness to average values. This, in turn, establishes an ideal clinical crown size, thereby preventing problems such as food impaction due to crowns of suboptimal dimensions. Additionally, it addresses concerns related to the physiological sliding of food during chewing, promoting optimal cleansing.

## 5. Conclusions

While acknowledging that statistical significance is achieved with a sufficient number of treated patients, we aim to underscore the numerous advantages of using this material in conjunction with the described technique for soft tissue augmentation encompassing both horizontal and vertical dimensions. Because of the inherent simplification of the procedure, primarily linked to its omission of membranes or other structures for protection, stabilization, and coverage, it is more straightforward than traditional techniques and accessible even to less experienced surgeons. The MTG technique, employing a high-consistency dermal matrix, has effectively thickened the specific peri-implant tissue biotype of patients, with a gain of 2.2 ± 0.38 mm reached (range 1.7–3.22 mm). This results in increased patient comfort and the recreation of an implant emergence profile more suited to the subsequently positioned prosthetic crown. Current research studies emphasize the importance of stabilizing peri-implant tissues as a critical factor in enhancing implants’ survival and success rates and minimizing undesired problems such as mucositis or peri-implantitis. Additionally, achieving a higher level of aesthetic mimicry can contribute to recreating an ideal soft tissue field. In light of all the above-mentioned indications, we can assert that this procedure is more sustainable from a biological perspective, while also considering the economic costs of regular prosthetic implant rehabilitations. The authors express their hope that this initial exploration serves as inspiration for numerous other colleagues and acts as a catalyst for a more comprehensive study of the material and technique. Despite being based on a small number of implants and patients, this technique has demonstrated remarkable effectiveness with consistent increments that stabilize vertically and horizontally over time.

The potential of this matrix will be expressed through future projects (split-mouth randomized studies) focused on a comparison between soft tissue augmentation using mean autologous connective tissue grafts and dermal matrices (the MTG technique).

## Data Availability

All the collected data are available in Dr. Minniti’s software database.

## Abbreviations

MTG: matrix tissue graft.
